# In vitro and in vivo probiotic assessment of *Leuconostoc mesenteroides* P45 isolated from *pulque*, a Mexican traditional alcoholic beverage

**DOI:** 10.1186/s40064-016-2370-7

**Published:** 2016-06-13

**Authors:** Martha Giles-Gómez, Jorge Giovanni Sandoval García, Violeta Matus, Itzia Campos Quintana, Francisco Bolívar, Adelfo Escalante

**Affiliations:** Departamento de Ingeniería Celular y Biocatálisis, Instituto de Biotecnología, Universidad Nacional Autónoma de México, Av. Universidad 2001, Col. Chamilpa, 62210 Cuernavaca, Morelos México; Departamento de Biología, Facultad de Química, Universidad Nacional Autónoma de México, Ciudad Universitaria, Coyoacán, 04510 Ciudad de México, México

**Keywords:** *Pulque*, *Leuconostoc mesenteroides*, Probiotic, Antimicrobial activity, Bacteriocin, Glucan-hydrolase

## Abstract

*Pulque* is a Mexican traditional alcoholic, non-distilled, fermented beverage produced by the fermentation of the sap, known as *aguamiel*, extracted from several maguey (*Agave*) species. *Pulque* has traditionally been considered a healthy beverage due to its nutrient content and also a traditional medicine for the treatment of gastrointestinal disorders and intestinal infections. During *pulque* fermentation, the development of acidity, alcohol and viscosity define its final sensorial properties, developing an enriched environment where dominant lactic acid bacteria (LAB), including diverse *Leuconostoc* species, are present. Because traditional *pulqu*e is consumed directly from the fermentation vessel, the naturally associated LAB are ingested and reach the human small intestine alive. Here, we report the in vitro and in vivo probiotic assessment of *Leuconostoc mesenteroides* strain P45 isolated from *pulque*. This isolated LAB species exhibited lysozyme, acid (pH 3.5) and bile salts (0.1 and 0.3 % oxgall) resistance. Antibacterial activity against the pathogens *Listeria monocytogenes*, enteropathogenic *Escherichia coli*, *Salmonella enterica* serovar Typhi and *S. enterica* serovar Typhimurium were observed in assays involving cell-to-cell contact, cell-free 2× concentrated supernatants and cell-to-cell contact under exopolysaccharide-producing conditions. The in vivo probiotic assessment showed an anti-infective activity of *L. mesenteroides* P45 against *S. enterica* serovar Typhimurium in challenged male and female BALB/c mice. Analysis of the available genome sequence of strain P45 allowed identified a pre-bacteriocin coding gene and six peptidoglycan hydrolase enzymes, probably involved in the antimicrobial activity of this strain. The results presented in this study support some potential microbial mechanisms associated with the beneficial effects on human health of this LAB involved in the fermentation of *pulque*.

**Electronic supplementary material:**

The online version of this article (doi:10.1186/s40064-016-2370-7) contains supplementary material, which is available to authorized users.

## Background

*Pulque* is a traditional alcoholic, non-distilled, fermented beverage produced by the fermentation of the sap known as *aguamiel,* extracted from several magueys (*Agave*) species such as *A. salmiana, A. atrovirens* and *A. mapisaga*. For its production, freshly collected *aguamiel* is transported to large open barrels where the fermentation takes place. The process is accelerated by the addition of a portion of previously produced *pulque*, and the fermentation time varies from a few hours to overnight, depending on whether the sap is collected at daybreak or dusk. The development of viscosity due to exopolysaccharide (EPS) synthesis, slight acidity and alcohol content are the main parameters used to determine the degree of fermentation. The process is static and performed under non-aseptic conditions; therefore, the populations of microorganisms involved in the fermentation are those naturally occurring during sap accumulation in maguey plants and those incorporated during collection, transport, inoculation and manipulation (Escalante et al. 2004, 2008, 2012; Lappe-Oliveras et al. 2008; Sanchez-Marroquin and Hope 1953).

*Aguamiel* and *pulque* have traditionally been considered as healthy beverages due to their nutrient content. They are a water substitute or alternative carbohydrate and protein source in places where drinking water is not available, of poor quality or where animal or vegetal proteins are scarce. Based on regular daily consumption, *pulque* is considered an important source of energy, vitamins and essential amino acids, such as lysine and tryptophan, which are deficient in the Mexican maize-based diet (Escalante et al. 2012; Lappe-Oliveras et al. 2008; Ortiz-Basurto et al. 2008; Sanchez-Marroquin and Hope 1953). Several of the health-promoting properties associated with the regular consumption of modest quantities of *aguamiel* or *pulque* are seen in rural populations (reviewed in Escalante et al. 2012).

Scientific evidence supporting the relationship between the microbial diversity present in *aguamiel* and *pulque* has shown the antimicrobial effects of both the sap used as substrate and the final fermented product against pathogenic bacteria such as *Salmonella enterica* serovar Typhimurium, *Staphylococcus aureus, Listeria monocytogenes*, *Shigella flexneri* and *S. sonnei* (Gómez-Aldapa et al. 2011). Although the results demonstrated antimicrobial activity during *pulque* fermentation, preventing the potential risk to consumers of contracting foodborne diseases by tested pathogens, the bactericidal effect was associated with the final alcohol content (~6 % v/v) and final pH (~4) but not with any specific bacterial activity.

*Aguamiel* produced by maguey species for *pulque* production contains large quantities of carbohydrates (~75 % of dry weight in *Agave masipaga* plants) and among them, 10 % by wt were identified as fructooligosaccharides, suggesting a potential prebiotic effect (Ortiz-Basurto et al. 2008). Moreover, the role of hydrocolloids as thermoprotector prebiotics (including fructooligosaccharides) in *aguamiel* was shown to enhance the growth of *Bifidobacterium bifidum* (Rodríguez-Huezo et al. 2007).

*Pulque* consumption for the treatment of gastrointestinal disorders and intestinal infections can also be explained by the possible probiotic activities associated with the presence of diverse lactic acid bacteria (LAB) such as *Leuconostoc citreum, L. kimchi. L. mesenteroides* and *Lactobacillus acidophilus* detected in fresh sap and during *pulque* fermentation (Escalante et al. 2004, 2008). To gain further evidence of the potential beneficial effects associated with LAB present in *aguamiel* and fermented *pulque*, we examined the probiotic properties of an LAB identified as *L. mesenteroides* strain P45 isolated from this traditional Mexican beverage. For that, we assessed its resistance to in vitro conditions simulating the gastrointestinal tract and the in vitro and in vivo antimicrobial activity against pathogenic bacteria.

## Results and discussion

### In vitro assessment of the resistance of *L. mesenteroides* P45 to gastrointestinal barriers

Screening for new potential probiotic LAB includes an assessment of their resistance to extreme antimicrobial environments associated with the human gastrointestinal tract, particularly their resistance to the lytic effect of lysozyme in human saliva when a probiotic preparation is consumed, and their resistance to the acid pH in the stomach, digestive enzymes such as pepsin, and bile salts secreted in the upper small intestine (García-Ruiz et al. 2014; Tripathi and Giri 2014). We assessed the resistance of strain P45 to in vitro lysozyme, acid pH and bile salts exposure. The results shown in Table 1 indicate that strain P45 was resistant to lysozyme, acid pH (2.5) and bile salt (0.3 and 1 %) exposure.

**Table 1:** In vitro resistance of *Leuconostoc mesenteroides* P45 to antimicrobial barriers

Microorganisms	Resistance to lysozyme (%)		Resistance to bile salts (%)		Resistance to pH 2.5 (%)
	30 min	120 min	0.3 %	1 %	
*L. mesenteroides* P45	89.56	70.71	100	100	74.98
*L. casei* Shirota	99.84	89.14	100	100	48.33

Percentage data shown corresponds to the average of three independent experiments conducted by triplicated

### Lysozyme resistance assay

Strain P45 showed ~90 and ~71 % resistance to 100 mg/L of lysozyme exposure at 37 °C after 30 and 120 min, respectively (Table 1). This assay condition for lysozyme resistance is considered extreme (García-Ruiz et al. 2014; Koll et al. 2008; Zago et al. 2011), as lysozyme is present in human biological fluids such as serum, saliva, human milk and mucus in the range of 1–13 mg/mL (Pushkaran et al. 2015). Assessment of potential probiotic LAB isolated from different sources, particularly for *Lactobacillus* species has shown that lysozyme resistance is a widespread property, showing moderate to high resistance (3.24–99.97 %) to 0.1–10 mg/mL of lysozyme in diverse lactobacilli isolated from saliva and subgingival sites (Koll et al. 2008) and from various types of cheese (Solieri et al. 2014; Zago et al. 2011).

In lactobacilli, resistance mechanisms to lysozyme have been associated with cell wall modifications, particularly with *N*- and *O*-substitutions in the peptidoglycan layer during stationary phase (Logardt and Neujahr 1975; Neujahr et al. 1973; Pushkaran et al. 2015) and lysozyme structure in the medium (García-Ruiz et al. 2014). No information describing resistance to lysozyme has been reported to *Leuconostoc* species. However, comparison between lysozyme resistance observed in strain P45 and available data for diverse lactobacilli isolates and the control probiotic bacteria included in this study (Table 1), allowed us to propose that treatment under conditions simulating lysozyme resistance to in vivo dilution by saliva observed for strain P45 is considered high to moderate after 30 and 120 min of incubation, respectively.

### Acid pH and bile salt resistance

Resistance to low pH and bile salt exposure are essential properties of potential probiotic microorganisms (Tripathi and Giri 2014). After oral consumption and transient exposure to lysozyme in the mouth, bacteria are exposed immediately to the extreme antimicrobial conditions in the stomach (pH between 1.5 and 3.0) (Zago et al. 2011). When probiotic bacteria reach the small intestine, they are additionally exposed to diverse antimicrobial conditions such as antimicrobial peptides, proteolytic enzymes and bile, including bile salts (ranging from 0.2 to 2 % w/v). Resistance to these conditions determines the number of viable cells which reach the small intestine to promote the strain-specific probiotic effects. Diverse authors have reported on acid pH and bile salts exposure, conditions that reflect the average time of food transit in the stomach and intestine in order to assess the potential probiotic properties of LAB isolated from fermented sources (Argyri et al. 2013). *L. mesenteroides* P45 showed 74.98 % resistance after exposure to acid pH (2.5) and 100 % resistance to bile salts both at 0.3 and 1 % after incubation for 24 h at 37 °C, whereas the reference probiotic strain showed only 48.33 % resistance to acid pH and 100 % survival against both of the bile salts concentrations assayed.

Previous reports on the assessment of the resistance to acid pH and bile salts concentrations for potential probiotic *L. mesenteroides* isolated from Algerian raw camel milk (exposition to pH 2–4 for 3 h at 37 °C), showed a reduction of viability of 21.17 % at pH 2 in one assayed strain, whereas the second was not able to survive to the acid treatment. Exposure of these strains to 0.5–2 % bile salts for 4 h at 37 °C showed 1.92–21.27 % resistance (Benmechernene et al. 2013). Assessment for acid pH and bile salts exposure in four isolates of *L. mesenteroides* from agave sap showed 40.9–49.12 % resistance to pH 2 for 3 h at 37 °C and 88–89 % resistance to 0.5 % bile salts for 4 h at 37 °C (Castro-Rodríguez et al. 2015). Resistance to acid pH and bile salts exposure observed for *L. mesenteroides* P45 and survival during a combined exposure to acid pH (2.5) + bile salts (0.3 %, 37 °C for 24 h) (Additional file 1) distinguished strain P45, which resisted remarkably to these conditions compared to the commercial probiotic assayed and other *L. mesenteroides* isolates.

### In vitro antibacterial assays against pathogenic bacteria

Antimicrobial activity against gastrointestinal pathogenic bacteria is one of the most important and desirable properties in potential probiotic bacteria (Soccol et al. 2012; Tripathi and Giri 2014). LAB can exert this antimicrobial activity by producing diverse fermentative metabolites with bactericidal or bacteriostatic activities such as lactic and acetic acids, fatty acids, hydrogen peroxide or diacetyl and antimicrobial proteins such as bacteriocins and peptidoglycan hydrolase enzymes (García-Cano et al. 2015; Perez et al. 2014).

**Fig. 1:**
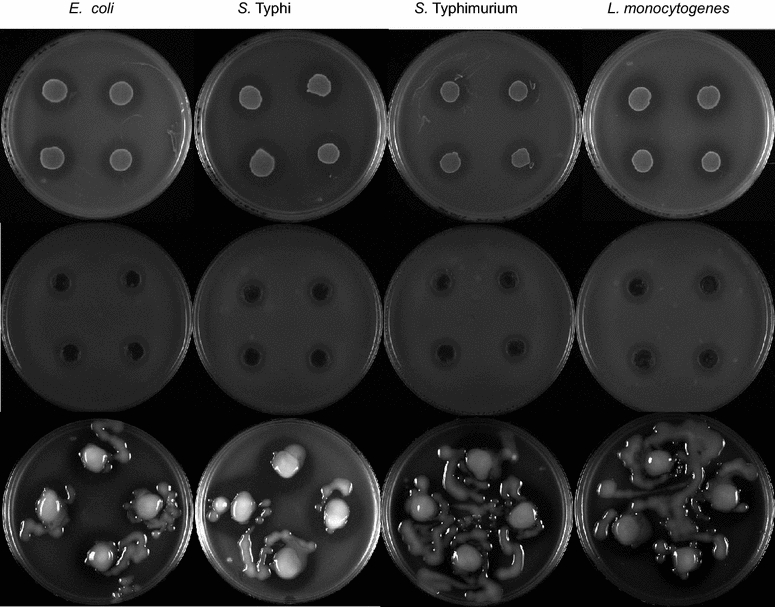
Antimicrobial activity of *L. mesenteroides* P45 against enteropathogenic *Escherichia coli*, *Salmonella enterica* serovar Typhimurium, *S. enterica* serovar Typhimurium and *L. monocytogenes*. *Upper panel* cell-to-cell antimicrobial effect of a lawn of strain P45. *Middle panel* 100 µL of cell-free, neutralized and 2× concentrated supernatant. *Bottom panel* cell-to-cell antimicrobial effect of a lawn of EPS-producing P45 grown on APT + 20 % sucrose. Mean ± SD inhibition zones (mm) observed for *upper panel* were: 11.75 ± 1.26, 11.25 ± 1.73, 9.25 ± 0.82, 10.5 ± 1.63, for each assayed bacteria, respectively. For *middle panel*: 7.5, 6.7 ± 0.5, 6.5 ± 0.58, 9.0 ± 1.15, respectively. Values of inhibition zone in EPS-producing cell-to-cell assays are omitted because the heterogeneous shape of the lawn of strain P45 (*bottom panel*)

Antimicrobial activity assays showed that *L. mesenteroides* P45 can inhibit the growth of enteropathogenic *E. coli* (EPEC)*, L. monocytogenes,* and *S. enterica* serovars Typhi and Typhimurium (Fig. 1). In vitro antimicrobial assays included supernatants with inhibition zones ranging from 6.5 to 8.5 mm (Fig. 1, middle panel). The results showed a higher bacteriostatic activity during cell-to-cell contact compared to diffusion assays with concentrated and neutralized supernatant cultures, suggesting that this antimicrobial activity is related to a cell-associated enzyme such as those found in the available draft genome sequence of this strain (Table 2) cell-to-cell contact between strain P45 and pathogenic bacteria showing inhibition zones ranging from 9.25 to 10 mm (Fig. 1, upper panel) and diffusion assays with cell-free concentrated and neutralized (pH 7.0).

**Table 2:** Relevant properties of proteins with possible antibacterial activity coded in the genome of *L. mesenteroides* P45

Protein	Relevant characteristics	GenBank accession	Length (amino acids)	Theoretical molecular mass (kDa)	Organisms/strains sharing identical proteins^a^
Pre-bacteriocin	Contains an Enterocin A immunity related protein motif	KGB49729	100	11.58	*L. mesenteroides* Wikim17 and KFRI-MG strains
Peptide ABC transporter ATP-binding protein.	ABC-type bacteriocin transporter	KGB49933	214	24.08	Not present in other organisms
Muramidase	Contains a LysM motif involved in binding to peptidoglycan	KGB50187	390	39.26	Not present in other organisms
1,4-β-*N*-acetylmuramidase	Lysozyme activity: Catalysis of the hydrolysis of the β-(1,4) linkages between *N*-acetylmuramic acid and *N*-acetyl-d-glucosamine residues in a peptidoglycan^b^	KGB50379	245	26.94	*L. mesenteroides* KFRI-MG
*N*-acetylmuramidase	Flagellum-specific peptidoglycan hydrolase FlgJ	KGB50418	212	23.93	*L. mesenteroides* subsp. *mesenteroides* ATCC 8293 and LbE16; *L. mesenteroides* Wikim17
1,4-β-*N*-acetylmuramidase	Cpl-1 lysin (also known as Cpl-9 lysozyme/muramidase) type enzyme. Bacterial cell wall endolysin which cleaves the glycosidic *N*-acetylmuramoyl-β-(1,4)-*N*-acetylglucosamine of glycan chain	KGB50910	363	39.72	Not present in other organisms
*N*-acetylmuramoyl-l-alanine amidase	Is an autolysin that hydrolyzes the amide bond between *N*-acetylmuramoyl and l-amino acids in certain cell-wall glycopeptides	KGB50968	287	31.73	Not present in other organisms
1,4-β-*N*-acetylmuramidase	Related to AtlA, an autolysin found in Gram-positive LAB that degrades bacterial cell walls by catalyzing the hydrolysis of 1,4-beta-linkages between *N*-acetylmuramic acid and *N*-acetyl-d-glucosamine residues	KGB51092	437	48.94	Not present in other organisms

^a^Information retrieved from the Conserved Protein Domain Family using the NCBI server. ^b^Information retrieved from the identical proteins tool using the NCBI server

Interestingly, some reports on the assessment of the probiotic potential of LAB isolates from diverse fermented sources have evaluated the production of EPS in the assayed strains, as it has been proposed that EPS production can positively affect the intestinal adhesion of probiotic bacteria (García-Ruiz et al. 2014), including some *Leuconostoc* strains isolated from diverse fermented products such as Korean kimchi (Ryu and Chang 2013) and water buffalo mozzarella cheese (de Paula et al. 2015).

Traditional *pulque* is a viscous product, this characteristic is mainly associated with the production of dextran and levan polymers synthesized from sucrose by diverse *Leuconosto*c species isolated from this beverage (Chellapandian et al. 1998; Torres-Rodríguez et al. 2014). Strain P45 produces an EPS when cultivated in APT or MRS broth supplemented with 20 % sucrose. Additionally, we evaluated the possible impact of the production of EPS by strain P45 on its in vitro antimicrobial activity. The results showed that EPS production has a positive impact on in vitro antimicrobial activity (Fig. 1, bottom panel) compared with assays performed with strain P45 grown in the absence of sucrose (Fig. 1, upper panel). However, the specific impact of EPS production on the antimicrobial properties of this bacterium is unclear.

### *In vivo* activity of *L. mesenteroides* P45 against the invasion of *S. enterica* serovar Typhimurium in mouse liver and spleen

**Fig. 2:**
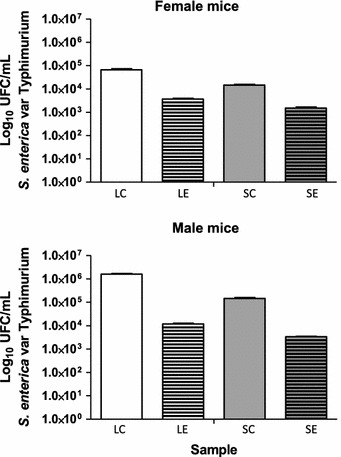
Anti-infective effect of *L. mesenteroides* P45 on *Salmonella enterica* serovar Typhimurium str^r^ in mouse liver and spleen. *LC* liver control, *LE* liver experimental, *SC* spleen control, *SE* spleen experimental. Values are the mean log_10_ CFU/mL count from liver and spleen samples obtained from nine mice per group

The anti-infective effect of the administration of *L. mesenteroides* P45 in BALB/c female and male mice against *S. enterica* serovar Typhimurium str^r^ was determined by analyzing the total CFU/mL in dissected liver and spleen of infected mice compared with control groups. The results showed that total CFU/mL of *Salmonella* decreased in livers and spleens dissected from experimental male and female mice (Fig. 2), but mean pathogen burden was significantly lower in female (Fig. 2a) than in male mice (Fig. 2b).

Protection against orally challenged *S. enterica* serovar Typhimurium strains on male BALB/c mice by oral feeding with *Lactobacillus rhamnosus* NH001 have been associated with the ability to confer immune enhancement. Results showed that infected and treated mice produced higher serum and intestinal titers of anti-*Salmonella* antibodies, higher ex vivo phagocyte capability, significant decrement in mean pathogen infection on liver and spleen and higher survival to infection respect the control group (Gill et al. 2001). Protection mechanisms have been also observed in BALB/c male mice fed with *Bifidobacterium lactis* strain HN109 isolated from yogurt, conferring protection against *Salmonella* infection by those mechanisms described above for *L. rhamnosus* NH001, including a reduced translocation of *Salmonella* in spleen and liver (Shu et al. 2000). Finally, colonization experiments with axenic C3/He/Oujco male mice with two strains of *Bifidobacterium* sp. isolated from infant stools protected challenged mice against infection of *S. enterica* serovar Typhymurium. Protection mechanisms were associated with colonization of the digestive tract of germ-free mice assayed and the efficient antimicrobial activity against infected *Salmonella* by established bifidobacteria (Lievin et al. 2000).

Additionally, infection of *Salmonella* between female and male mice is known to be associated with sex hormones, which regulate the immune response between sexes, resulting in differences in immune cell activation, infiltration and cytokine production during injury and infection (Bird et al. 2008; Yamamoto et al. 1991). The lower infection level in female mice has been attributed to estrogen, which has been associated with resistance to infection through stimulation of the immune system against pathogens such as *Mycobacterium marinum*. In males, testosterone has been proposed to reduce host resistance to infection by *Leishmania* by suppressing the bactericidal functions of macrophage cells and inhibiting the clonal proliferation of B cells (Yamamoto et al. 1991).

The metabolomic and transcriptomic analysis of the feces and liver using a murine typhoid infection model revealed an important impact of *Salmonella* infection on host metabolism, particularly on host hormone signaling pathways such as eicosanoid, steroid, and primary bile acid biosynthesis and sugar metabolism. Disruption of these pathways may support *Salmonella* infection (e.g., eicosanoids control important functions such as vasoconstriction, platelet aggregation and the immune response) (Antunes et al. 2011). The reduced infection level observed in the liver and spleen in our work suggest that administration of *L. mesenteroides* strain P45, fed for 7 days before *Salmonella* infection, possibly stimulates the host immune response by alleviating the impact of infection.

### Identification of proteins with antimicrobial activity from the draft genome of *L. mesenteroides* P45

The analysis of the available draft genome of *L. mesenteroides* P45 allowed us to identify the coding sequences of a pre-bacteriocin (GenBank accession KGB49933) and six peptidoglycan hydrolase (PGH) enzymes (Table 2): β-(1,4)-*N*-acetylmuramidase (KGB50379, KGB50910, KGB51092), *N*-acetylmuramidase (KGB5041) and *N*-acetylmuramoyl-l-alanine amidase enzymes (KGB50968), with molecular weights ranging from 23.93 to 48.94 kDa. Among them, the pre-bacteriocin and four PGH enzymes did not have identical proteins in other organisms according to the identical proteins tool available on the NCBI server (Table 2).

Bacteriocins are peptides which have antibacterial activity. Bacteriocins produced by *Leuconostoc* species have been reported and characterized, and they are all Class II bacteriocins (small, <10 kDa, heat-stable, non-lanthionine-containing peptides (Perez et al. 2014)), including leuconin A (produced by species of *L. gelidum, L. pseudomenteroides*) (Makhloufi et al. 2013), leuconin B (produced by species of *L. mesenteroides*, *L. carnosum*) (Benmechernene et al. 2013) and the circular bacteriocins (lucocyclicin Q produced by *L. mesenteroides*) (Gabrielsen et al. 2014). PGHs are enzymes that can hydrolyze glycosidic bonds or peptides found in the bacterial cell wall peptidoglycan layer and are also involved in cellular functions such as growth, division and autolysis (autolysins). PGHs are classified according to the peptidoglycan bond type that they hydrolyze, as follows: (1) *N*-acetylmuramidases (muramidases, hydrolyzing the β-(1-4) bond between MurNAc and GlcNAc), including lysozymes and lytic transglycosylases; (2) *N*-acetylglucosaminidase (glucosaminidases, hydrolyzing the β-(1-4) bond between GlcNAc and MurNAc); (3) *N*-acetylmuramoyl-l-alanine amidase [amidases, hydrolyzing the bond between the lactyl group of MurNAc and the α-amino group of l-Ala (the first amino acid of the lateral peptidic chain of the peptidoglycan layer)]; and (4) peptidases, including endopeptidases and carboxypeptidases, which hydrolyze a variety of peptidoglycan bonds (Chapot-Chartier and Kulakauskas 2014; García-Cano et al. 2011). The production of PGH has been reported in LAB such as *Lactobacillus casei, L. helveticus, L. plantarum, L. pentosus, Lactococcus lactis, Pediococcus pentosaceus* and *P. acidilactici* (Chapot-Chartier and Kulakauskas 2014; García-Cano et al. 2011, 2015).

Previous reports on the identification and characterization of PGHs produced by *Leuconostoc* species include the study of the PGH Mur in *L. citreum* 22R and the 1L10, an autolysin produced by *L. mesenteroides* and *L. mesenteroides* subsp. *mesenteroides.* These PGHs are involved in cheese ripening processes which include *Leuconostoc* sp. together with lactic-acid producing *Lactococcus* sp. (Cibik et al. 2001; Cibik and Chapot-Chartier 2000). PGHs found in the draft genome of *L. mesenteroides* P45 (Table 2) isolated from *pulque* require further characterization in order to determine their possible role in the autolytic or in the antimicrobial activities against pathogenic bacteria.

## Conclusions

Traditional Mexican fermented *pulque* beverage is considered a nutritional and health-promoting beverage, particularly in the treatment of gastrointestinal disorders. As traditional *pulque* is consumed without any treatment affecting the bacterial viability, living LAB are consumed, reaching the human intestine and offering benefits to the health of the consumer. The assessment of the probiotic potential of *L. mesenteroides* P45 isolated from *pulque* showed that this strain is highly resistant to the antimicrobial barriers assayed in vitro, particularly exposure to acid pH and bile salts. This strain exhibited important in vitro antibacterial activity against Gram-positive and Gram-negative bacteria possibly associated with a combined effect of a bacteriocin and a PGH coded in the genome of this bacterium. Interestingly, production of EPS from sucrose apparently promotes the in vitro antimicrobial activity against pathogenic bacteria assayed. Administration of living strain P45 to BALB/c mice causes a decrement in the infection with *S. enterica* serovar Typhimurium both in female and male mice. The data reported in this study provide scientific evidence suggesting several microbial mechanisms which may underlie the beneficial effects associated with the consumption of living LAB from *pulque*.

The isolation and characterization of LAB isolated from non-dairy environments such as traditional fermented sources, and their incorporation in the formulation of foods and beverages, particularly drinks based on fruit and cereals is considered as a global trend (Soccol et al. 2012). The beneficial effects of *L. mesenteroides* P45 make it a good candidate for its incorporation in this kind of functional products.

## Methods

### Bacterial strains and culture conditions

*Leuconostoc mesenteroides* strain P45 was selected from a set of LAB isolated from *pulque* collected from the town of Huitzilac in Morelos State (19°1′42″N, 99°16′4″W, 2530 meters above sea level). These isolates were assayed for potential probiotic properties (Additional file 1). Among them, strain P45, survived remarkably compared the other isolates also identified as *Leuconostoc* sp. The complete whole-genome shotgun project of strain P45 was deposited at DDBJ/EMBL/GenBank under the accession number JRGZ00000000 (Riveros-Mckay et al. 2014).

For routine culture conditions, *L. mesenteroides* P45 was grown at 30 °C in MRS or APT (DIFCO) broth or on plates. EPEC *E. coli* 2348/69, *S. enterica* serovar Typhi ATCC9992, and *S. enterica* serovar Typhimurium ATCC14028 were grown on nutritive broth (DIFCO) or agar-nutritive plates; *L. monocytogenes* was grown on nutritive broth (DIFCO) or agar-nutritive plates supplemented with 1 % yeast extract (DIFCO). These bacteria were provided by the Culture Collection of the Faculty of Chemistry, National Autonomous University of Mexico (CFQ World Data Centre for Microorganism number 100), with the exception of EPEC *E. coli* 2348/69 (Treviño-Quintanilla et al. 2007) and streptomycin-resistant *S. enterica* serovar Typhimurium L1334 (str^r^), which were kindly provided by Dr. Edmundo Calva and Dr. Víctor Bustamante, Instituto de Biotecnología, UNAM (IBT-UNAM). These bacteria were grown in nutrient broth (DIFCO) supplemented with 1 % yeast extract (DIFCO) at 37 °C.

### Assessment of lysozyme, bile salt and acid resistance

The in vitro simulation activity of saliva was assessed as described previously (Solieri et al. 2014). Lysozyme (Sigma-Aldrich) was tested at 100 mg/L in a sterile electrolyte solution (SES (g/L): 0.22 of CaCl_2_, 6.2 of NaCl, 2.2 of KCl, 1.2 of NaHCO_3_). 10 mL of APT were inoculated with a fresh colony of strain P45 grown in APT agar, centrifuged at 5000×*g* at 4 °C and resuspended in the same volume of SES containing lysozyme. The cell suspensions were adjusted to a final cell density corresponding to the spectrophotometric optical density at 600 nm (OD_600nm_), equivalent to 10^9^ CFU/mL. Aliquots of the bacterial suspension were exposed to lysozyme for 30 or 120 min. The OD_600nm_ was determined, a tenfold dilution was performed, and the CFU/mL were enumerated by plating on APT agar. Bacterial suspensions in SES without lysozyme were included as controls.

Bile salt and acid resistance were assessed as reported previously with slight modifications (Sahoo et al. 2015; Solieri et al. 2014). For bile salt resistance assays, a cell suspension of strain P45 was prepared as described above. One mL of the desired OD_600nm_ was inoculated in 9 mL of APT broth supplemented with 0.1 and 0.3 % bile salt (oxgall, Oxoid), and the suspension was incubated al 37 °C for 24 h. The viability was determined as for the lysozyme resistance assays. For the acid resistance assays, a cell suspension (1 mL), obtained as above, was used to inoculate 9 mL of APT broth adjusted to pH 2.5 with 6.0 N HCl, and the suspension was incubated at 37 °C for 5 h without agitation. A sample of 1 mL was removed and serially diluted in phosphate buffered saline solution (PBS, 0.8 % NaCl, 0.121 % K_2_HPO_4_, 0.034 % KH_2_PO_4_, pH 7.4), and the resultant CFU/mL was determined on APT plates. The controls were performed with the desired cell suspension in APT broth without bile salts and in non-acidified APT broth. *Lactobacillus casei* Shirota isolated from a commercial probiotic beverage was used as a positive control in experiments for lysozyme, acid pH and bile salt resistance.

### In vitro antibacterial assays

The qualitative in vitro antibacterial activity of *L. mesenteroides* P45 was tested against pathogenic bacteria as follows: an aliquot of 0.1 mL of a cell suspension containing 1 × 10^9^ CFU/mL of an overnight culture of strain P45 grown in APT was dropped onto fresh APT in quadruplicate and incubated overnight at 37 °C. The resultant growth lawn was overlaid with 5 mL of nutritive soft agar containing 0.5 mL of an overnight culture of pathogenic bacteria (EPEC *E. coli, S. enterica* serovar Typhi, *S. enterica* serovar Typhimurium and *L. monocytogenes*) adjusted to an optical density of OD_600nm_ = 0.2. The plates were then incubated overnight at 37 °C and scored for antibacterial activity by measuring each zone of inhibition with a millimeter ruler around the growth lawn. The control experiments were performed without strain P45 (Additional file 1).

### In vivo anti-infective activity of *L. mesenteroides* P45 against *S. enterica* serovar Typhimurium

*L. mesenteroides* P45 was assayed for in vivo anti-infective effect against streptomycin-resistant *S. enterica* serovar Typhimurium strain L1334 (str^r^) in female and male BALB/c mice acquired from the animal facility center at IBT-UNAM. The experiment was performed as described previously with slight modifications (Chiu et al. 2007). Previous reports describing assays in mice for the in vivo assessment of the anti-infective activity of potential probiotic LAB included animal groups ranging from 6 (Hudault et al. 1997) to 10–12 (Chiu et al. 2007) or 12–16 animals (Tsai et al. 2005). Our control and experimental groups consisted of 9 mice. Strain P45 was grown as described above, centrifuged (5000×*g*, for 5 min at 4 °C), and resuspended in 1 mL of PBS, and the cell density was adjusted to 2 × 10^9^ CFU/mL per dose. The mice were fed for seven consecutive days. On the 8  days, each animal was inoculated with a single 0.2 mL dose of 1 × 10^7^ UFC/mL of *S. enterica* serovar Typhimurium strain L1334 in PBS. The control and experimental groups were maintained with water and food at a constant temperature (25 °C). After 3 days, the mice were sacrificed by cervical dislocation, and the spleens and livers were dissected aseptically, mixed with sterile demineralized water (up to 5 mL) and homogenized with sterile glass beads using a vortex. The cell suspensions were serially diluted in sterile saline and plated on agar LB supplemented with 100 mg/mL str (Sigma-Aldrich). The total CFU/mL was determined after incubation at 37 °C for 24 h.

### Statistical analysis

In order to determine if the observed differences between the growth of *S. enterica* serovar Typhimurium strain L1334 in the control and experimental groups’ organs (liver and spleen) were significant (P < 0.05), an analysis of variance (ANOVA) and the multiple comparison tests of Tukey´s Honestly Significant Difference (HSD) were performed using the XLSTAT program (www.xlstat.com).

### Identification of antimicrobial proteins coded in the genome of *L. mesenteroides* P45

From the available draft genome sequence of *L. mesenteroides* P45 at DDBJ/EMBL/GenBank (Riveros-Mckay et al. 2014), we retrieved the coding sequences for a pre-bacteriocin and six hydrolytic enzymes with possible antimicrobial activity: 1,4-β-*N*-acetylmuramidase, *N*-acetylmuramidase and *N*-acetylmuramoyl-l-alanine amidase.

## Additional file

10.1186/s40064-016-2370-7 **Screening of probiotic properties of LAB isolated from pulque.** In vitro resistance of isolated LAB against combined exposition of acid pH and bile salt for 24 h. Qualitative in vitro antimicrobial activity of selected LAB 1 against *L. monocytogenes, S. enterica* serovar Typhimurium, *S. enterica* serovar Typhi and EPEC *E. coli*. Summary of in vitro qualitative antimicrobial activity of LAB isolated from pulque against pathogenic bacteria.
