# Supplementary material for: In vitro and in vivo probiotic assessment of Leuconostoc mesenteroides P45 isolated from pulque, a Mexican traditional alcoholic beverage
Source: Springerplus. 2016 Jun 13;5(1):708. doi: 10.1186/s40064-016-2370-7 (PMC4906094; doi:10.1186/s40064-016-2370-7)
Supplement: Supplementary file 1 — 10.1186/s40064-016-2370-7 Screening of probiotic properties of LAB isolated from pulque. In vitro resistance of isolated LAB against combined exposition of acid pH and bile salt for 24 h. Qualitative in vitro antimicrobial activity of selected LAB 1 against L. monocytogenes, S. enterica serovar Typhimurium, S. enterica serovar Typhi and EPEC E. coli. Summary of in vitro qualitative antimicrobial activity of LAB isolated from pulque against pathogenic bacteria. [file 40064_2016_2370_MOESM1_ESM.pdf]

Additional Table 1. *In vitro* resistance of isolated LAB from *pulque* fermentation against combined exposition of acid pH and bile salt for 24 h.

**Strain**	**Colony morphology**	**Identity**[a]	**Resistance to combined acid pH 2.5 + 0.3% bile salt exposition (%)**	
			30 °C	37 °C
P4	Diplococci	*Leuconstoc kimchii*	29.5	0
P9	Diplococci	*Leuconstoc durionis*	21.2	14.0
P31	Diplococci	*Leuconstoc kimchii*	14.8	0
P45	Diplococci	*Leuconstoc mesenteroides*	27.1	24.6
P49	Diplococci	*Leuconstoc kimchii*	14.1	0
P59CH	Diplococci	*Leuconstoc kimchii*	0	13.7
P59G	Diplococci	*Leuconstoc kimchii*	25.0	0
3H38	Diplococci	*Leuconstoc kimchii*	9.9	15.1
3H52	Diplococci	*Leuconstoc kimchii*	19.2	0
6H15	Diplococci	*Leuconstoc durionis*	17.5	13.5
6H39	Diplococci	*Leuconstoc durionis*	22.0	12.1
6H74	Diplococci	*Leuconstoc kimchii*	23.9	13.7
L. casei Shirota		*L. casei* Shirota	27.7	22.0

[a]Based on 16S rDNA sequence analysis as described previously (Escalante et al. 2004; Escalante et al. 2008)

Additional Figure 1. Qualitative *in vitro* antimicrobial activity of selected LAB from Additional Table 1 against *L. monocytogenes*

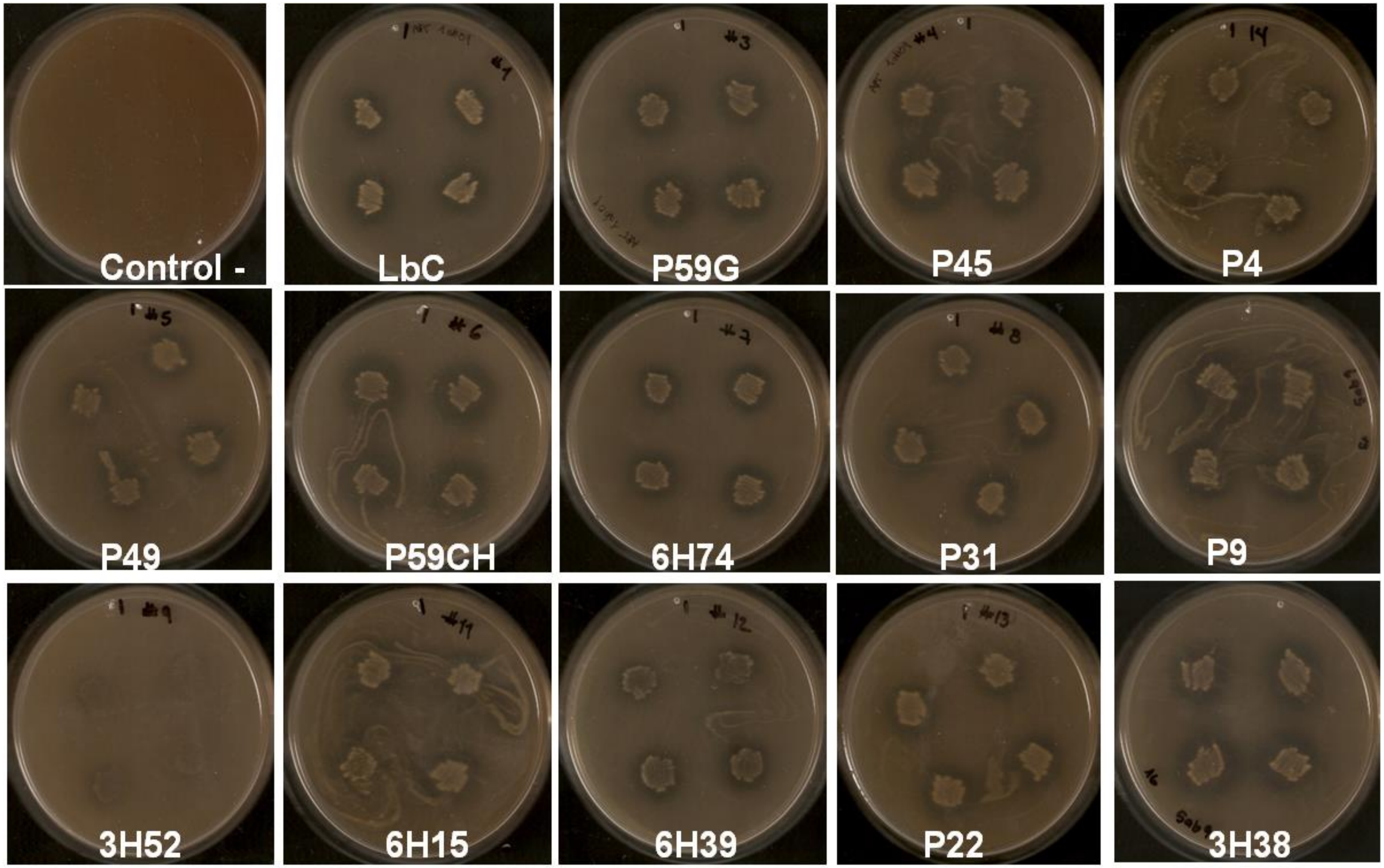

Control -, APT plate covered with pathogenic microorganism adjusted to an optical density at OD_{600nm} = 0.2, LbC, *L. casei* Shirota

Additional Figure 2. Qualitative *in vitro* antimicrobial activity of selected LAB from Additional Table 1 against *S. enterica* serovar Typhimurium

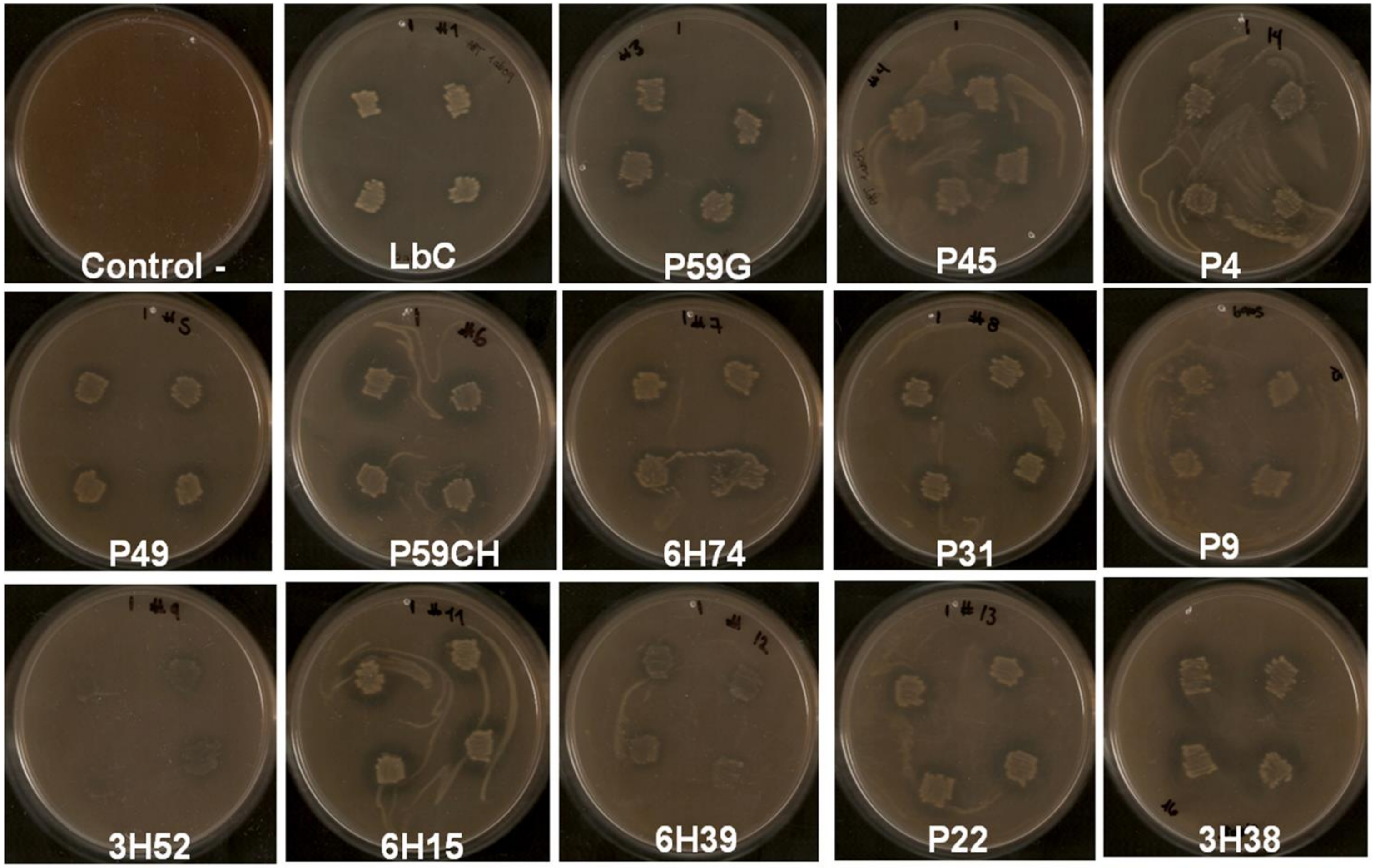

Control -, APT plate covered with pathogenic microorganism adjusted to an optical density at OD_{600nm} = 0.2, LbC, *L. casei* Shirota

Additional Figure 3. Qualitative *in vitro* antimicrobial activity of selected LAB from Additional Table 1 against *S. enterica* serovar Typhi.

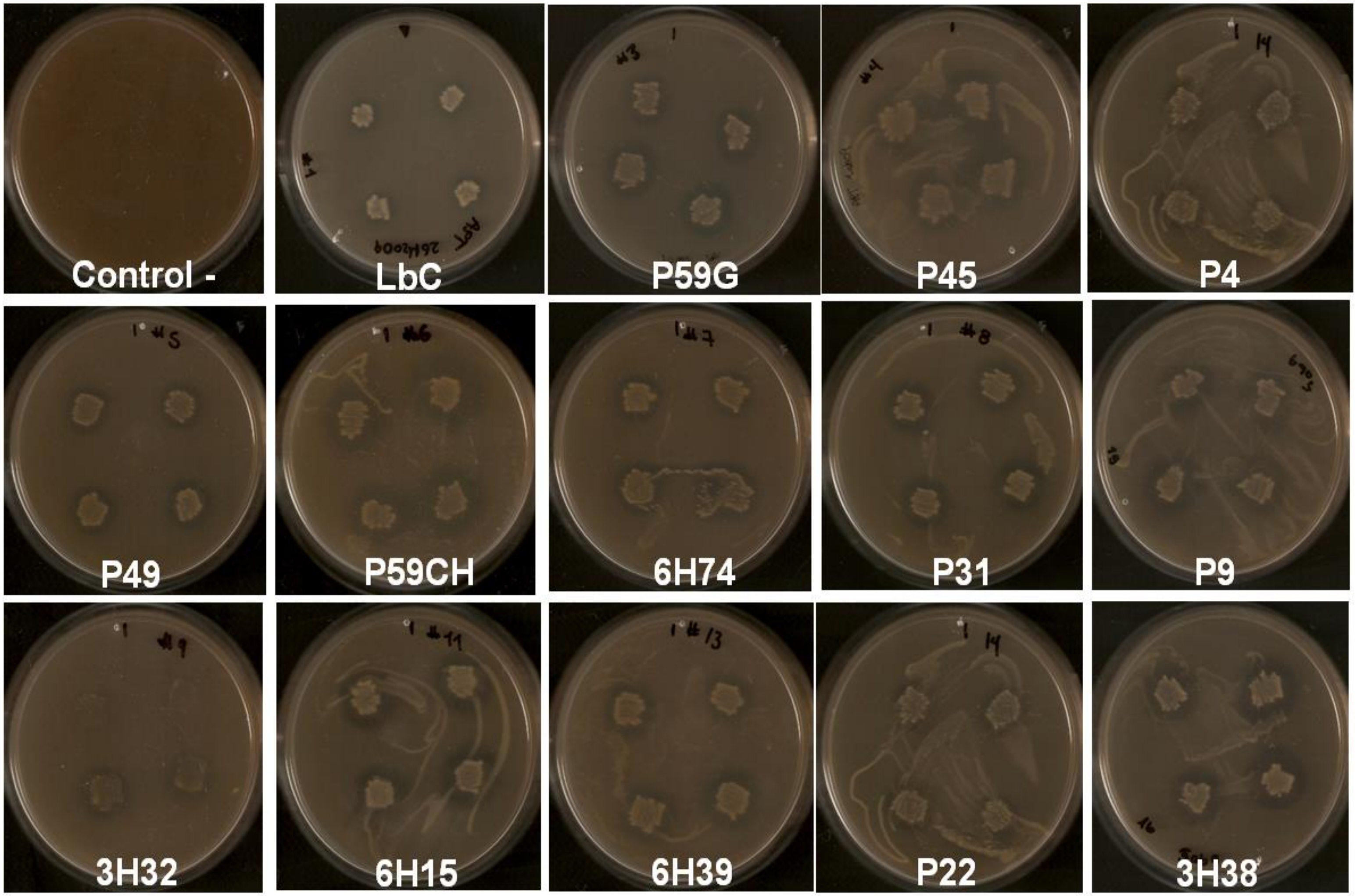

Control -, APT plate covered with pathogenic microorganism adjusted to an optical density at OD_{600nm} = 0.2, LbC, *L. casei* Shirota

Additional Figure 4. Qualitative *in vitro* antimicrobial activity of selected LAB from Additional Table 1 against EPEC *E. coli*.

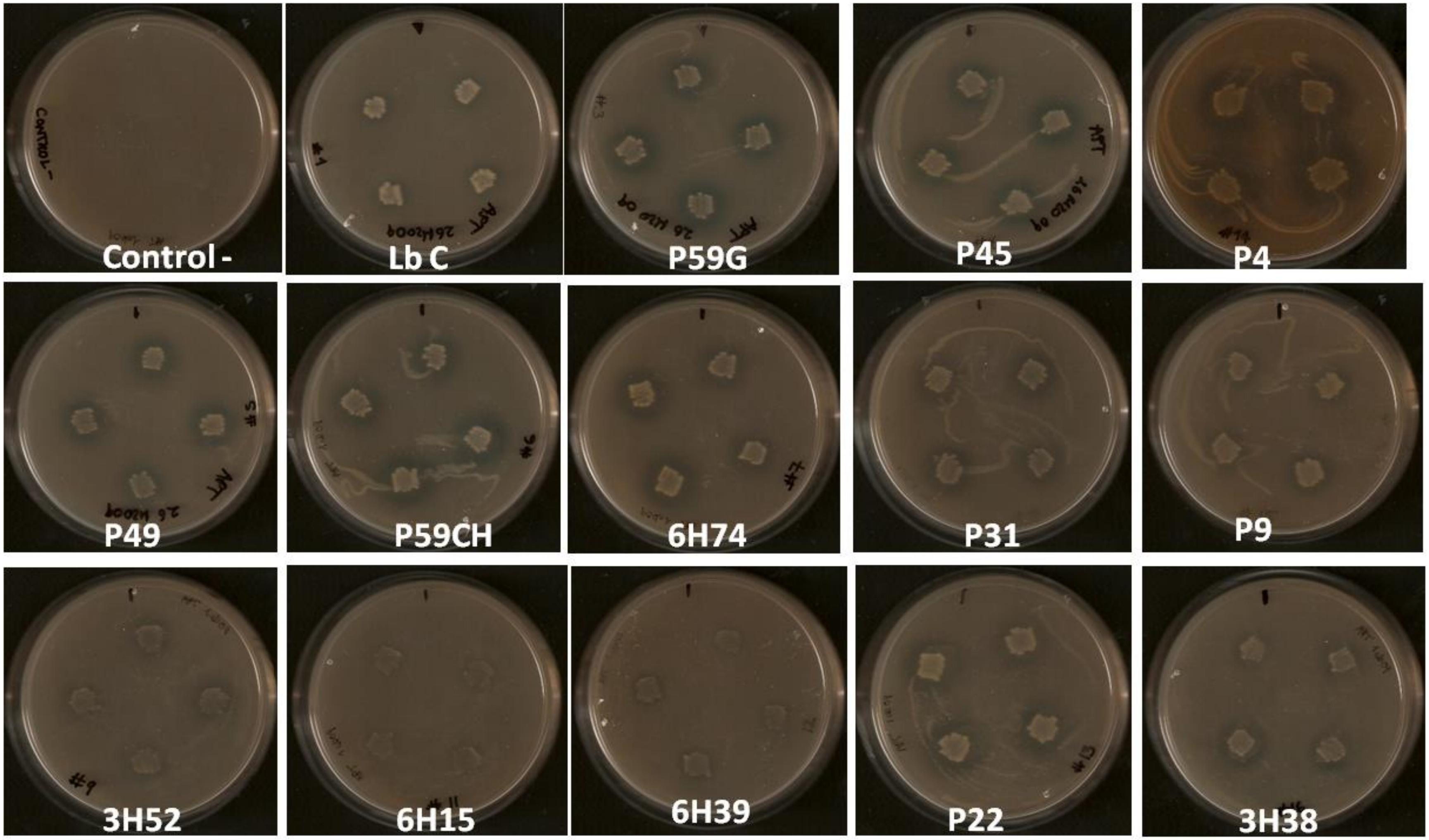

Control -, APT plate covered with pathogenic microorganism adjusted to an optical density at OD_{600nm} = 0.2, LbC, *L. casei* Shirota

Additional Table 2. Summary of *in vitro* qualitative antimicrobial activity of LAB isolated from *pulque* against pathogenic bacteria.

**Isolated strain**	**Antimicrobial activity**			
	***E. coli* EPEC**	***L. monocytogenes***	***S. enterica* var Typhi**	***S. enterica* var Typhimurium**
P4	++++	++	++	++
P9	++	++++	+++	++
P22	+++	++	+	++
P31	+++	+++	+++	+++
P45	+++	++++	++++	++++
P49	++	++	++	+++
P59CH	+++	++++	++++	++++
P59G	+++	+++	+++	++++
3h38	++	+++	+++	++
3H52	+	+	++	+
6H15	+	+	+++	+++
6H39	+	+	+	+
6H74	++	++	++	++
L. casei Shirota	+	++	+	+
